# Dexmedetomidine Enhances Autophagy *via* α2-AR/AMPK/mTOR Pathway to Inhibit the Activation of NLRP3 Inflammasome and Subsequently Alleviates Lipopolysaccharide-Induced Acute Kidney Injury

**DOI:** 10.3389/fphar.2020.00790

**Published:** 2020-06-24

**Authors:** Tianyuan Yang, Xiujing Feng, Yuan Zhao, Haiyang Zhang, Hailin Cui, Mian Wei, Haotian Yang, Honggang Fan

**Affiliations:** Heilongjiang Key Laboratory for Laboratory Animals and Comparative Medicine, College of Veterinary Medicine, Northeast Agricultural University, Harbin, China

**Keywords:** acute kidney injury, dexmedetomidine, autophagy, NLRP3 inflammasome, α2-AR/AMPK/mTOR pathway

## Abstract

**Background:**

Acute kidney injury (AKI) is a severe complication of sepsis; however, no effective drugs have been found. Activation of the nucleotide-binding domain-like receptor protein 3 (NLRP3) inflammasome is a major pathogenic mechanism of AKI induced by lipopolysaccharide (LPS). Autophagy, a process of intracellular degradation related to renal homeostasis, effectively restricts inflammatory responses. Herein, we explored the potential protective mechanisms of dexmedetomidine (DEX), which has confirmed anti-inflammatory effects, on LPS-induced AKI.

**Methods:**

AKI was induced in rats by injecting 10 mg/kg of LPS intraperitoneally (i.p.). Wistar rats received intraperitoneal injections of DEX (30 µg/kg) 30 min before an intraperitoneal injection of LPS. Atipamezole (ATI) (250 µg/kg) and 3-methyladenine (3-MA) (15 mg/kg) were intraperitoneally injected 30 min before the DEX injection.

**Results:**

DEX significantly attenuated renal injury. Furthermore, DEX decreased activation of the NLRP3 inflammasome and expression of interleukins 1β and 18. In addition, autophagy-related protein and gene analysis indicated that DEX could significantly enhance autophagy. Finally, we verified the pharmacological effects of DEX on the 5′-adenosine monophosphate-activated protein kinase (AMPK)/mechanistic target of rapamycin (mTOR) pathway. Atip and 3-MA significantly reversed the protective effects of DEX.

**Conclusions:**

Our results suggest that the protective effects of DEX were mediated by enhanced autophagy *via* the α_2_-adrenoreceptor/AMPK/mTOR pathway, which decreased activation of the NLRP3 inflammasome. Above all, we verified the renal protective effects of DEX and offer a new treatment strategy for AKI.

## Introduction

Sepsis, a clinical syndrome that occurs in response to infection, is characterized by systemic hyperinflammation, dysregulation of the immune response, and multiple organ failure (Zarjou and Agarwal, 2011; Binkowska et al., 2015). Acute kidney injury (AKI) is one of the main effects observed with multiple organ dysfunction in patients suffering from sepsis; indeed, sepsis is responsible for 50% of all cases of AKI (Bagshaw et al., 2009; Plotnikov et al., 2018). The pathogenesis of severe AKI involves microcirculatory dysfunction, inflammation, and bio-energetic adaptive responses. In particular, inflammation plays an important role in the pathogenesis of AKI (Murugan et al., 2010; Zarbock et al., 2014). Because of its heterogeneous pathological processes, currently the only viable solution for AKI is renal replacement therapy (Gatward et al., 2008). However, the high price of renal replacement therapy and scarcity of kidney sources makes AKI a heavy burden to healthcare systems. At present, no drugs have been approved by the United States Food and Drug Administration for the treatment of AKI (Chen et al., 2019), and no optimal treatments are available for AKI resulting from sepsis (Wu et al., 2015). Thus, the mechanisms and treatment of AKI still need further elucidation.

Lipopolysaccharide (LPS) present within the cell wall of Gram-negative bacteria is one of the main causes of sepsis (Remick et al., 2000; Bhargava et al., 2013). As such, injection of LPS is widely used in animal studies to establish AKI models (Doi et al., 2009). LPS, a notable source of sepsis, plays an important role in the pathogenesis of AKI by causing excessive inflammatory responses and subsequent escalation of oxidative stress, renal hypoperfusion, and severe kidney injury (Chen et al., 2018). LPS can combine with toll-like receptor 4 (TLR4) to induce an intracellular response by recruiting transcription factors such as nuclear factor κB (NF-κB) in the nucleus, followed by secretion of chemokines and cytokines that regulate inflammatory processes and immune responses (Heinbockel et al., 2018). LPS is a typical member of pathogen-associated molecular patterns (PAMPs). The NLRP3 inflammasome functions as an innate sensor of several PAMPs and damage-associated molecular patterns (DAMPs), and acts an imperative mediator of inflammatory responses in various models of AKI (Shen et al., 2016). Previous studies reported a close relationship between the NLRP3 inflammasome and inflammatory responses during exacerbation of AKI (Kim et al., 2013; Wen et al., 2018). NLRP3 inflammasome activation is associated with caspase activation recruitment domain (ASC) and caspase-1, and promotes caspase-1 cleavage (Schroder and Tschopp, 2010). Activation of the NLRP3 inflammasome has been shown to regulate the maturation and excretion of inflammatory cytokines, especially interleukin 1β (IL-1β) and IL-18, leading to an inflammatory response (Sun et al., 2013). In addition, recent studies demonstrated a relationship between activation of the NLRP3 inflammasome and mitochondrial function (Deng et al., 2019). As mitochondrial membranes are involved in NLRP3 activation, the proximity of NLRP3 to mitochondria is a vital indicator of kidney injury (Lei et al., 2012). Some previous studies reported moderate renal-protective effects in NLRP3-knockout mice (Kim et al., 2013; Kim et al., 2018b). Therefore, the NLRP3 inflammasome is an important therapeutic target for preventing inflammatory responses associated with AKI.

Dexmedetomidine (DEX) is a selective α_2_-adrenoreceptor (α_2_-AR) agonist with sedative, analgesic, and anti-anxiety effects (Shen et al., 2017). In addition, several animal studies have noted antioxidant, anti-apoptosis, and anti-inflammatory effects of DEX, although some of the molecular pathways remain unclear (Kutanis et al., 2016; Wang et al., 2019). Many studies have demonstrated that DEX can decrease endotoxin-induced upregulation of inflammatory molecules and attenuate renal function associated with AKI (Lai et al., 2009; Liang et al., 2017; Qiu et al., 2018). In addition, recent studies have shown that DEX can decrease expression of the NLRP3 inflammasome and provide protective effects against renal injury (Kim et al., 2018a; Yin et al., 2018). However, the mechanism by which DEX downregulates the NLRP3 inflammasome to reduce inflammation has not been clearly identified.

Autophagy has been recognized as essential for maintaining cellular homeostasis and stress responses (Lenoir et al., 2016). Autophagy serves as a degradation system by which intracellular pathogens, damaged or long-lived proteins, and dysfunctional organelles are encased into autophagosomes and eliminated in lysosomes (Marino and Lopez-Otin, 2004; Wang et al., 2018a). Several studies have shown that autophagy can block activation of the NLRP3 inflammasome, subsequently inhibiting IL-1β and IL-18 (Shi et al., 2012b; Hong et al., 2019). Autophagy, which plays a protective role in the pathological processes of renal tubular injury, has been widely studied. The mechanistic target of the rapamycin (mTOR) pathway has been acknowledged as a key inhibitor of autophagy in response to a variety of intracellular disorders. As an upstream negative regulator of mTOR, 5′ adenosine monophosphate-activated protein kinase (AMPK) also plays a vital role in anti-inflammatory processes (White et al., 2015). However, it is unclear whether the mechanism by which DEX protects the kidney is related to autophagy. Hence, this potential relationship remains to be explored.

Our findings, which provide evidence that DEX has renal protective effects, explore the underlying mechanism by which DEX enhances autophagy in response to AKI induced by sepsis. The results may provide a novel therapeutic strategy for AKI.

## Material and Methods

### Animals and Treatment

Thirty-six adult male Wistar rats were obtained from the Second Affiliated Hospital of Harbin Medical University (Harbin, China). Rats weighed 180-220g and were housed in a room that had a 12h light and dark cycle (lights on from 6:00-18:00) with temperature 20 ± 2°C and humidity 45%-55% for one week to adapt to the environment. Rats were divided randomly groups (3 per cage) and were fed ad libitum with standard food and fresh tap water. All experimental procedures in this study met the requirements of the Animal Experimental Committee of Northeast Agricultural University and complied with the National Institutes of Health Guide for the Care and Use of Laboratory Animals.

The Wistar rats were randomly divided into six groups (n=6 per group):

1. CON group was injected intraperitoneally with saline.
2. CON+DEX group was injected intraperitoneally with DEX (30 µg/kg, American Pfizer).
3. LPS group was injected intraperitoneally with LPS (10 mg/kg, L2630-100MG, Sigma-Aldrich, USA) for 4h to establish the animal model of sepsis-induced AKI as described previously (Feng et al., 2019).
4. LPS+DEX group was injected intraperitoneally with DEX (30 µg/kg) 30 min before treatment with LPS (10 mg/kg).
5. LPS+DEX+ATI group was injected intraperitoneally with Atipamezole (ATI) (250 μg/kg, American Pfizer), an α2-receptor inhibitor, 30 min before treatment with DEX (30 μg/kg), and injected intraperitoneally with DEX 30 min before treatment with LPS (10 mg/kg).
6. LPS+DEX+3-MA group was injected intraperitoneally with 3-MA (15 mg/kg) 30 min before treatment with DEX (30 μg/kg), and injected intraperitoneally with DEX 30 min before treatment with LPS (10 mg/kg).

All rats initially received inhalation anesthesia with 1.5% isoflurane (Yipin Pharmaceutical, Co., Ltd., Hebei, China) and were sacrificed after 4h. Then, we collected blood, urine, and kidney samples.

### Biochemical Indexes Analysis

The blood samples were collected quickly by heart puncture and kept at room temperature for 30 min. The serum was obtained by centrifugation at 3500 rpm for 10 min at 4°C from blood samples. The collected serum was measured for serum creatinine (Scr) and blood urea nitrogen (BUN) using a UniCel DxC800 Synchron (Beckman, USA). Urine samples were collected by bladder puncture for the analysis of kidney injury molecule-1 (KIM-1) using an ELISA kit (R&D Sytstems, Minneapolis, MN).

### Histopathological Analysis

For analysis, kidneys were fixed in 4% paraformaldehyde, embedded in paraffin, and cut into 5μm thickness sections. The sections were stained with hematoxylin and eosin stain. Images of stained tissues were visualized and captured using a light microscopy (BX-FM; Olympus Corp, Tokyo, Japan). The kidney histological scores were quantified by ten renal cortex regions from every section (400x magnification). The percentages of tubules that showed tubular dilatation and vacuolization, interstitial edema, brush border defect, and inflammatory cell infiltration were scored as follows: 0 = none, 1 = 0 - 20%, 2 = 20 - 50%, 3 = 50 - 70%, 4 = more than 70% (Brooks et al., 2009). And observers used a double-blinded approach to evaluate scores.

### Immunohistochemistry Analysis

The 3μm thick paraffin-embedded kidney sections were dewaxed, and then dehydrated using graded concentrations of alcohol. To inhibit endogenous peroxidase, the sections were incubated with 3% H_2_O_2_. The sections were microwaved in citric acid for 15 min, and then treated with goat serum for 15 min at room temperature. Afterwards, the sections were incubated in blocking solution with primary antibody at 4°C overnight. After washing with PBS 3 times, the secondary antibody was added and immunostaining was performed using a DAB horseradish peroxidase color development kit (Beyotime, China), and then sections were counterstained with hematoxylin and made transparent with xylene. Finally, sections were observed with the PD37 type microscope (Olympus,Japan). Under 400 × magnification, pictures were taken in 5 random fields. Primary antibodies were used at the following dilutions: IL-1β diluted 1:100 (WL02257, Wanlei, Shenyang, China); IL-18 diluted 1:100 (WL01127, Wanlei, Shenyang, China).

### ELISA Assay

The levels of IL-1β (H002) and IL-18 (H0015) in serum were detected with an ELISA kit according to the manufacturers’ instructions (Nanjing Biotechnoloy Co., Ltd., Nanjing, China).

### Real-Time Polymerase Chain Reaction (RT-PCR) Analysis

Total RNA was isolated from the kidney tissue with Trizol reagent (Invitrogen, Carlsbad, CA, American) according to the manufacturer’s instructions, and reverse transcribed into cDNA using the PrimeScript RT reagent kit (DRR037A; Takara, Dalian, China). Then, quantitative real-time PCR detection of RNA copies were performed on a Light Cycler^®^ 480 II Detection System (Roche) using IQ SYBR Supermix reagent (Bio-Red, San Diego, CA). The relative expression levels were normalized to GAPDH and analyzed by the 2^-ΔΔCt^ method. The primers for the detection of target mRNA are listed in **Table 1**.

**Table 1:** Primer sequence in this study.

Genes	Sequence (5′ - 3′)
p62	(F) CCCGTCTACAGGTGAACTCC (R) CTGGGAGAGGGACTCAATCA
Beclin1	(F) GTTGCCGTTATACTGT (R) TTTCCACCTCTTCTTTGA

### Western Blot

Frozen kidney tissues were cut into small pieces and lysed with RIPA lysis buffer (Beyotime Biotechnology, Shanghai, China). Phenylmethanesulfonyl fluoride (PMSF) (Beyotime Biotechnology, Shanghai, China) was added, and the tissue was homogenized through a Tissue Grinding instrument (Shanghai Jingxin Industrial Development Co., Ltd., Shanghai, China), and then centrifuged at 3000 rpm for 10 min at 4°C to collect the supernatant. Protein concentrations were quantified by a BCA Protein Assay kit (Beyotime Biotechnology, Shanghai, China). Equal amounts of protein sample were separated by standard Tris-glycine SDS-PAGE gel electrophoresis, and then transferred to polyvinylidene difluoride (PVDF) membranes. After blocking with 5% skimmed milk for 2h at room temperature, the PVDF membranes were incubated with primary antibodies at 4°C overnight. Primary antibodies and dilutions were as follows: AMPKα (WL02254, Wanlei, Shenyang, China) diluted 1:500; p-AMPKα2(Ser173) (bs-5575R, Bioss, Beijing, China) diluted 1:1000; mTOR (A2245,ABclonal,Wuhan,China) diluted 1:1000; p-mTOR (Ser2448) (AP0094, ABclonal, Wuhan, China) diluted 1:1000; LC3 (A5202, Bimake, Houston, American) diluted 1:1000; Beclin (D40C5, Cell Signaling Teghnology, American) diluted 1:1000; p62 (WL02385, Wanlei, Shenyang, China) diluted 1:500; NLRP3 (WL02635, Wanlei, Shenyang, China) diluted 1:1500, IL-1β (WL02385, Wanlei, Shenyang, China) diluted 1:500; IL-18 (WL01127, Wanlei, Shenyang, China) diluted 1:1000; caspase-1 (WL02996a, Wanlei, Shenyang, China) diluted 1:750; cleaved-caspase-1 (WL03450, Wanlei, Shenyang, China); ASC (A11433, ABclonal, Wuhan, China); GAPDH (WL01114, Wanlei, Shenyang, China). After washing five times with Tris-buffered saline containing Tween (TBST), the membranes were incubated with 1: 20000 horseradish peroxidase-conjugated goat anti-rabbit IgG secondary antibody (ZB-2301, ZSGB-BIO, Beijing, China) or anti-mouse IgG secondary antibody (ZB-2305, ZSGB-BIO, Beijing, China) at room temperature for 2h and then washed with TBST, followed by development using ECL reagent (WLA003, Wanlei, Shenyang, China), captured by the Amersham Imaher 600 software (GE, American), and analyzed using Image J software.

### Transmission Electron Microscopy

The number of autolysosome and ultrastructural changes were detected by transmission electron microscopy. Kidney tissues were cut into about 1 mm × 1 mm × 1 mm pieces and placed in 4% glutaraldehyde at 4°C for 12h. The samples were post-fixed in 1% osmic acid for 90 min and washed by 0.1M PBS 3 times for 15 min each. After that, the samples were dehydrated in a graded series of ethanol (50%, 70%, 90%, 100%) and embedded in epoxy resin. The ultrathin sections were prepared and then stained with uranyl acetate and lead citrate. The sections were observed with transmission electron microscopy (Hitachi HT7700, Tokyo, Japan).

### Immunofluorescence Staining

The 3μm thick paraffin sections were deparaffined, rehydrated, and prepared for immunofluorescence assays according to a standard protocol. The sections were incubated with primary antibodies as follows: anti-NLRP3 (WL02635, Wanlei, Shenyang, China) diluted 1:200 and anti-TOM20 (A19403, ABclonal, Wuhan, China) diluted 1:100 overnight at 4°C. After being washed with PBS 3 times, the sections were incubated with secondary antibody. The sections were washed with PBS 3 times again and then sealed with coverslips. Fluorescence images were acquired with a Nikon Eclipse Ni inverted microscope (TE2000; Nikon, Tokyo, Japan).

### Statistical Analysis

Data were expressed as mean ± SD (standard deviation). All statistical analyses were performed using the PASW statistics 18 software (SPASS, IL, USA). Comparisons among multiple groups with measurement data obeying normal distribution were conducted using one-way analysis of variance (ANOVA), and comparisons between two groups were made using the least square method (LSD). Graphs were made using GraphPad Prism5 (San Diego, California). *p* < 0.05 was considered statistically significant. Statistical differences were considered to be extremely significant when *p* < 0.01.

## Results

### DEX Improved Renal Function in Rats With Sepsis

**Figure 1:**
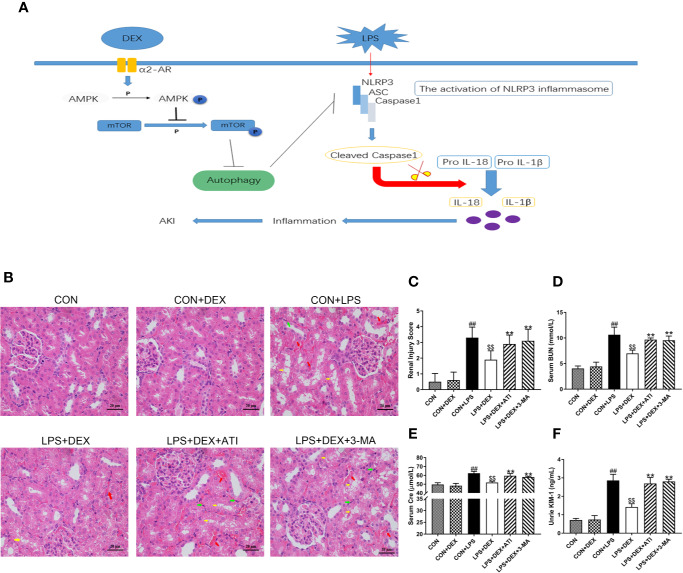
DEX improved renal damage induced by LPS-induced AKI. **(A)** Sepsis-induced AKI is established by intraperitoneally injecting LPS (10mg/kg) into rats. The activation of NLRP3 inflammasome caused inflammatory responses that led to renal injury. DEX enhances autophagy through the α2-AR/AMPK/mTOR pathway to inhibit inflammation and protect the kidney. **(B)** Represented images of H&E staining (× 400) in the renal cortex. Red arrow indicates hemorrhage, yellow arrow indicates vacuolar degeneration, and black arrow indicates infiltration of intertubular inflammatory cells. Scale bars = 20μm. **(C)** The histopathological score of kidney damage. **(D)** The level of serum BUN in rats. **(E)** The level of serum Cre in rats. **(F)** The level of urine KIM-1 in rats. Data are expressed as mean ± SD (n = 6). *^##^p* < 0.01 compared with CON group. *^$$^p* < 0.01 compared with CON+LPS group. ^**^p < 0.01 compared with LPS+DEX group. CON: control; DEX: dexmedetomidine; LPS: lipopolysaccharide; ATI: atipamezole; 3-MA: autophagy inhibitor.

To investigate whether DEX improved the kidney function of rats with sepsis, we assessed levels of renal function indicators: blood urea nitrogen (BUN, **Figure 1D**), creatinine (CRE, **Figure 1E**), and kidney injury molecule-1 (KIM-1, **Figure 1F**). All three indicators were significantly increased in the LPS group compared with the control (CON) group. However, treatment with DEX significantly decreased levels of all three markers, indicating that DEX improved the renal function of rats with sepsis. In addition, treatment with the α_2_-AR inhibitor ATI or autophagy inhibitor 3-MA abolished the protection elicited by DEX against sepsis-induced renal dysfunction. The observed insignificant difference between CON and CON+DEX groups suggested that DEX had no effect on normal rats.

### DEX Ameliorated Pathology in Rats With Sepsis

To determine the impact of DEX on renal tissue injury, we detected the pathological changes in the kidney by microscopy (**Figures 1B, C**). Normal kidney structures were observed in the CON group. After LPS injection, kidney tissues displayed renal tubular epithelial cell vacuolar degeneration, renal tubular cavity expansion, hemorrhage, and infiltration of intertubular inflammatory cells. However, DEX ameliorated this pathological damage. Furthermore, ATI and 3-MA reversed the effects of DEX.

### DEX Ameliorated Inflammatory Response by Reducing NLRP3 Inflammasome and Inflammatory Cytokines in Rats With Sepsis

**Figure 2:**
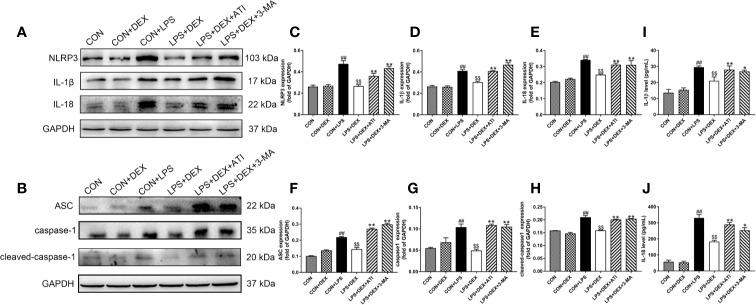
DEX ameliorates inflammatory responses in kidney tissue and serum induced by LPS. **(A)** Protein levels of NLRP3, IL-1β, and IL-18. **(B)** Protein levels of ASC, caspase-1, cleaved-caspase-1. **(C–E)** The protein expression of NLRP3, IL-1β, and IL-18 were normalized to the level of GAPDH protein. (**F–H)** The protein expression of ASC, caspase-1, cleaved-caspase-1 were normalized to the level of GAPDH protein. **(I)** The content of IL-1β in serum. **(J)** The content of IL-18 in serum. Data are expressed as mean ± SD (n=6). *^##^p* < 0.01 compared with CON group. *^$$^p* < 0.01 compared with CON+LPS group. ^*^p < 0.05, ^**^p < 0.01 compared with LPS+DEX group. CON: control; DEX: dexmedetomidine; LPS: lipopolysaccharide; ATI: atipamezole; 3-MA: autophagy inhibitor.

**Figure 3:**
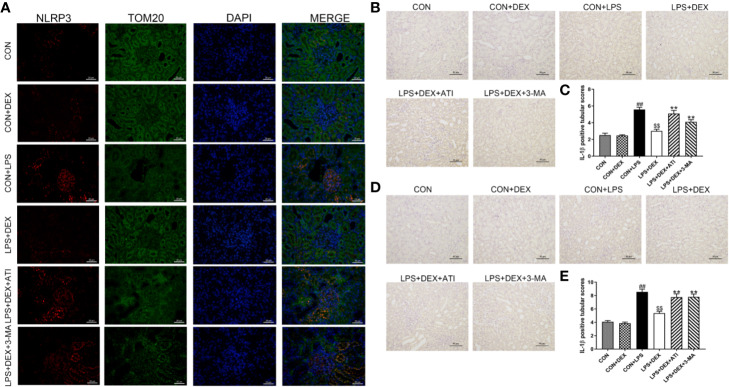
**(A)** The positive expression of NLRP3 in kidney tissue was observed by immunofluorescence staining under a laser scanning confocal microscope. The NLRP3 cells were marked in red, the mitochondrial membranes were stained with TOM20 in green, and the nuclear was stain with DPAI in blue. Scale bars = 50 μm. **(B)** Immunohistochemistry analysis of IL-1β in kidney tissue. **(C)** Quantitative analysis of IL-1β in kidney tissue. **(D)** Immunohistochemistry analysis of IL-18 in kidney tissue. **(E)** Quantitative analysis of IL-18 in kidney tissue. Data are expressed as mean ± SD (n=6). *^##^p* < 0.01 compared with CON group. *^$$^p* < 0.01 compared with CON+LPS group. ^**^p < 0.01 compared with LPS+DEX group. CON: control; DEX: dexmedetomidine; LPS: lipopolysaccharide; ATI: atipamezole; 3-MA: autophagy inhibitor.

To determine whether sepsis was successfully established, we examined changes in serum levels of inflammatory factors (**Figures 2I, J**). Enzyme-linked immunosorbent assay results revealed significantly upregulated serums levels of IL-1β and IL-18 level in response to LPS, while DEX obviously ameliorated these changes. However, ATI and 3-MA reversed the effect of DEX. By further evaluating the inflammatory response of renal tissue (**Figures 2A–H**), we found that LPS significantly increased expression of IL-1β, IL-18, NLRP3, ASC, caspase-1, and cleaved-caspase-1, which were all downregulated by DEX. Moreover, ATI and 3-MA could eliminate the effects of DEX. Immunohistochemical analysis to confirm the localization of inflammatory cytokines in the kidney tissue indicated the presence of IL-18 and IL-1β near the renal tubule, as well as significant increases in LPS, LPS+DEX+ATI, and LPS+DEX+3-MA groups. However, DEX could reverse these changes. Moreover, according to calculated IOD values, immunohistochemical results were consistent with western blot results (**Figures 3B–E**). To evaluate the localization of NLRP3 and mitochondria, NLRP3 and the mitochondrial membrane protein TOM20 were stained for co-immunofluorescence microscopy. The results showed that LPS increased NLRP3 expression, whereas DEX decreased NLRP3 expression, and ATI and 3-MA reversed the effect of DEX. Moreover, NLRP3 clearly localized with mitochondria (**Figure 3A**).

### DEX Ameliorate LPS-Induced NLRP3 Inflammasome Activation by Regulating Autophagy

**Figure 4:**
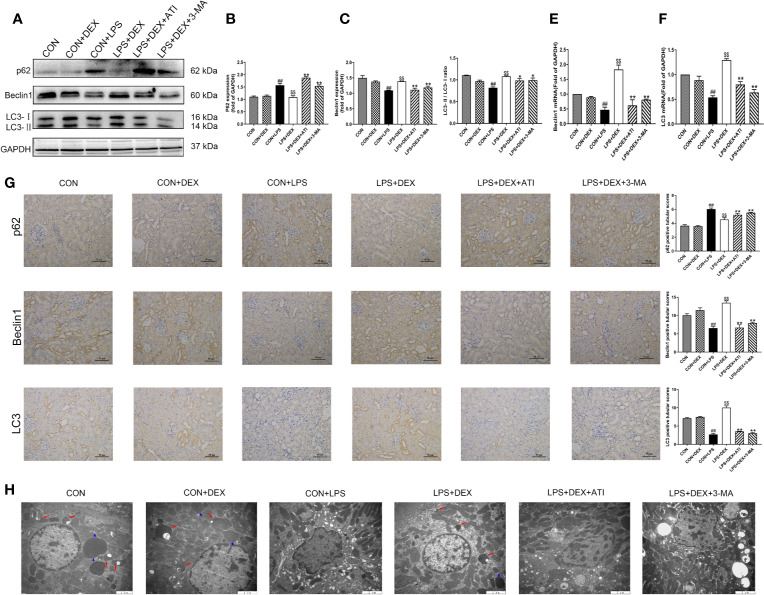
DEX enhanced autophagy. **(A)** Protein levels of p62, Beclin1, LC3. **(B–D)** The protein expression of p62, Beclin1, LC3 were normalized to the level of GAPDH protein. **(E)** mRNA expression of Beclin1. **(F)** mRNA expression of LC3. **(G)** Immunohistochemistry analysis and quantitative analysis of p62, Beclin1, LC3 in kidney tissue. **(H)** Represented ultrastructure by transmission electron microscopy in kidney tissue. Red arrow indicated autolysosome and blue arrow indicated lysosome. Scale bars = 2 μm. Data are expressed as mean ± SD (n=6). *^##^p* < 0.01 compared with CON group. *^$$^p* < 0.01 compared with CON+LPS group. ^*^p < 0.05, ^**^p < 0.01 compared with LPS+DEX group. CON: control; DEX: dexmedetomidine; LPS: lipopolysaccharide; ATI: atipamezole; 3-MA: autophagy inhibitor.

To confirm the role of autophagy in DEX-regulated NLRP3 activation, we examined the autophagy-related proteins microtubule-associated protein light chain 3 (LC3), beclin-1, and p62. Our results showed that LPS decreased the expression of LC3-II and beclin-1, but increased expression of p62. With DEX intervention, the LC3-II/LC3-I ratio and expression of beclin-1 were significantly increased, while expression of p62 was decreased. However, ATI and 3-MA could suppress the effects of DEX, as they reduced the LC3-II/LC3-I ratio and beclin-1 expression, and increased p62 expression (**Figures 4A–D**). Immunohistochemical analysis of LC3, beclin-1, and p62 indicated expression levels consistent with western blot results. Moreover, autophagy was observed to occur near the renal tubule (**Figure 4G**). Ultrastructural observations of the kidney tissue indicated that LPS caused a large number of pathological changes, such as shrunken nuclei, mitochondrial disruption, and fuzzy mitochondrial cristae. DEX ameliorated these pathological changes and increased the number of autolysosomes compared with the LPS group. Using the autophagy inhibitor 3-MA, we observed obvious decreases in the number of autolysosomes, which were replaced by intracellular damage. Intervention with ATI produced the same effects as 3-MA (**Figure 4H**). Upon measuring transcription levels, we found that LPS significantly decreased LC3 and beclin-1 mRNA expression. After DEX treatment, LC3 and beclin-1 mRNA expression were obviously upregulated. However, both 3-MA and ATI could suppress the effect of DEX (**Figures 4E, F**).

### DEX Ameliorate LPS-Induced NLRP3 Inflammasome Activation by Enhancing Autophagy *via* AMPK/mTOR Pathway

**Figure 5:**
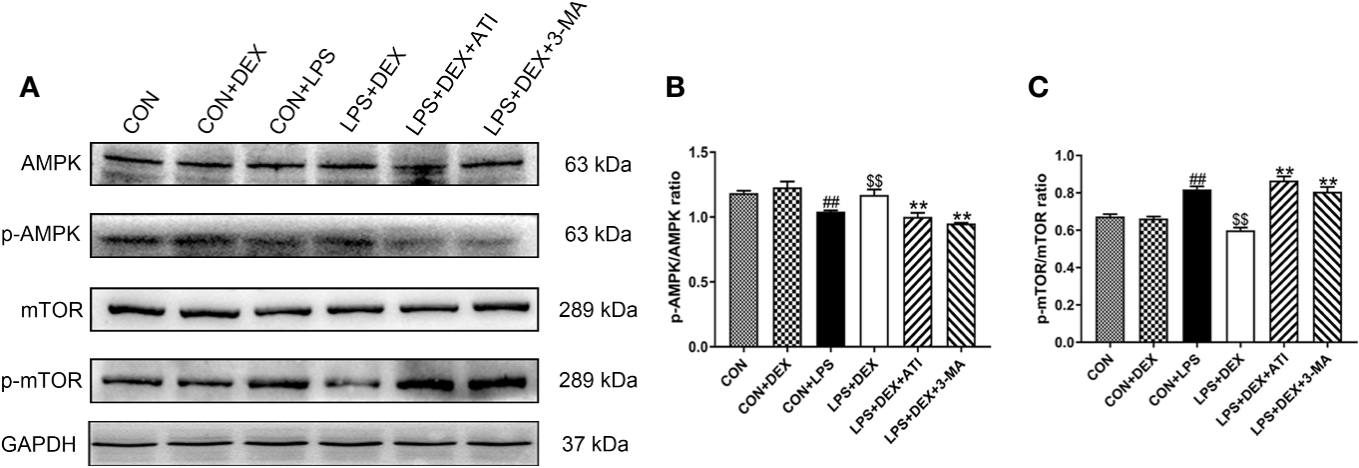
Dex enhanced autophagy *via* AMPK/mTOR pathway. **(A)** Protein levels of AMPK, p-AMPK, mTOR and p-mTOR. **(B)** The protein expression of p-AMPK was normalized to the level of AMPK protein. **(C)** The protein expression of p-mTOR was normalized to the level of mTOR protein. Data are expressed as mean ± SD (n=6). *^##^p* < 0.01 compared with CON group. *^$$^p* < 0.01 compared with CON+LPS group. ^*^p < 0.05, ^**^p < 0.01 compared with LPS+DEX group. CON: control; DEX: dexmedetomidine; LPS: lipopolysaccharide; ATI: atipamezole; 3-MA: autophagy inhibitor.

To verify the correlation between the AMPK/mTOR pathway and the pharmacological effects of DEX, we examined expression of AMPK, phosphorylated AMPK (p-AMPK), mTOR, and p-mTOR (**Figures 5A–C**). Our western blot results showed that after treatment with LPS, expression of p-AMPK tended to decrease while expression of p-mTOR increased. However, DEX reversed these effects of LPS, and ATI and 3-MA could block the effects of DEX.

## Discussion

AKI complicated by sepsis is a clinical syndrome associated with high mortality and morbidity. As a lack of optimal treatments is responsible for these results, effective treatments are urgently needed. In the present study, we first verified the establishment of our AKI model and the protective effects of DEX in the kidney. Next, we examined the inflammatory response induced by activation of the NLRP3 inflammasome. We then confirmed that NLRP3 inflammasome activation and inflammatory cytokines could be inhibited by DEX-enhanced autophagy. Finally, we determined that DEX enhanced autophagy *via* the α2-AR/AMPK/mTOR pathway.

AKI induced by sepsis is closely associated with excessive inflammatory responses and severe renal impairment. Indeed, the kidney is one of the earliest and most frequently affected organs during sepsis (Bagshaw et al., 2008; Jin et al., 2020). Therefore, early intervention can prevent further exacerbation of AKI and more serious damage caused by sepsis. According to previous studies, an early stage of AKI was established 4 h after intraperitoneal injection (Tunctan et al., 2018; Feng et al., 2019). Histopathological and biochemical analyses are classical techniques to evaluate kidney function. With LPS intervention, we observed infiltration of intertubular inflammatory cells, vacuolar degeneration of the tubular lining epithelium, tubular dilatation, and hemorrhaging. Biochemical indicators also reflected renal dysfunction, including significantly increased levels of BUN, CRE, and KIM-1. KIM-1 reportedly regulates renal function recovery and tubular degeneration, and thus serves as an indicator of tubular injury (Ichimura et al., 2008). Our results show that AKI can be initiated by LPS stimulation. However, pretreatment with DEX prevented the occurrence of disorders both pathologically and biochemically. Similar to the results of a previous study (Feng et al., 2019), our findings demonstrate that DEX exerted a renal protective capacity against AKI induced by LPS. However, potential underlying mechanisms still need to be explored.

LPS-induced inflammatory responses are the cause of severe renal dysfunction (Gomez et al., 2014). As a starting point for treatment, inhibition of inflammatory responses may be an effective strategy for sepsis-induced AKI. A previous report indicates that LPS can increase NLRP3 activation in AKI animal models (Chunzhi et al., 2016). Recently, more extensive studies demonstrated that activation of the NLRP3 inflammasome mediates maturation and secretion of IL-1β and IL-18, a process that prominently contributes to AKI (Shen et al., 2016; Sogawa et al., 2018). As an important proinflammatory factor, IL-1β not only stimulates the kidney to produce aggressive inflammatory responses, but can cause severe renal re-absorption disorders (Wang et al., 2015; Schett et al., 2016; Liu et al., 2019a). As a marker, IL-18 is more than 90% sensitive and specific to diagnosed AKI, and high expression of IL-18 can eventually lead to tubular damage (Parikh et al., 2004; Shi et al., 2012a). Our results showed that LPS can promote NLRP3 inflammasome activation, as well as IL-1β and IL-18 expression. Furthermore, serum levels of IL-1β and IL-18 indicated the presence of a widespread inflammatory response. A previous study confirmed that damage associated with LPS-induced AKI occurred in renal tubular epithelial cells (Li et al., 2019; Lu et al., 2019), consistent with our immunohistochemistry results. Thus, stimulation with LPS can induce activation of the NLRP3 inflammasome and promote an excessive inflammatory response in the kidney. Other previous studies indicated that mitochondrial dysfunction also plays a vital role in NLRP3 inflammasome activation. Both mitochondrial reactive oxygen species and membrane proteins are involved in activation of the NLRP3 inflammasome (Kim et al., 2018b; Chung et al., 2019). In our study, we observed that NLRP3 localized with TOM20, a mitochondrial protein, as well as ultrastructural damage in mitochondria. These results suggest a close relationship between NLRP3 and mitochondria. However, the process of NLRP3 inflammasome activation needs further exploration. Regardless, DEX pretreatment could inhibit activation of the NLRP3 inflammasome and largely alleviate the inflammatory response induced by LPS.

Autophagy, a highly dynamic process of intracellular degradation, is closely related to the elimination of damaged proteins and dysfunctional organelles (Marino and Lopez-Otin, 2004). Accumulating evidence indicates that NLRP3 inflammasome activation is inhibited by enhanced autophagy (Wong et al., 2018; Torp et al., 2019), which can also reduce inflammatory cytokines associated with LPS-induced AKI (Zhao et al., 2019). LC3 protein is imperative for initiating the formation of autophagosomal membranes. LC3-II arises from a combination of LC3-I and phosphatidyl ethanolamine upon initiation of autophagy (Li et al., 2020). The ratio of LC3-II/LC3-I expression, indicating the conversion of LC3-I to LC3-II, is a crucial indicator of autophagy (Chen et al., 2010). In addition, beclin-1 is essential for regulating autolysosome formation (Deretic et al., 2013). Expression of p62 protein, an autophagy adapter protein that binds to ubiquitinated protein aggregates and LC3-II (Guo et al., 2020), is contrary to that of LC3 and beclin-1. Surprisingly, the autophagy response to LPS in our study differed from previous studies. The low autophagy level presented in our study was consistent with Radovan Vasko’s study (Vasko et al., 2013). However, after LPS intervention, this lack of autophagy enhanced NLRP3 inflammasome activation and inflammatory cytokine expression; perhaps this dosage of LPS destroys autophagy. Although the reason for this discrepancy in results is unclear, it may be caused by differences in experimental models. Autophagy-related genes LC3 and beclin-1 were decreased after LPS intervention; thus, the effect of LPS on autophagy at a transcriptional level was confirmed. LPS pretreatment could obviously suppress autophagy and induce intracellular injury. However, DEX significantly restored autophagy in our study, consistent with the results of Oh et al. (2019). According to these results, we confirmed that DEX can restore the lack of autophagy induced by LPS.

It is difficult to determine whether the observed reductions in injury elicited by DEX were related to its role in increasing autophagy. To solve this puzzle, we selected the autophagy inhibitor 3-MA to verify this relationship. As a class-III phosphoinositide 3 kinase inhibitor, 3-MA is widely used to inhibit autophagy, frequently at a dosage of 15 mg/kg (Wu et al., 2015; Bao et al., 2018; Zhao et al., 2019). Under the influence of 3-MA, autophagy was significantly decreased, whereas renal injury and expression of inflammatory cytokines, such as NLRP3, IL-1β, and IL-18, were increased. Hence, autophagy can alleviate LPS-induced renal injury by downregulating the inflammatory response elicited by NLRP3 inflammasome activation. The dosage of atipamezole (250 µg/kg), a complete α_2_-AR antagonist, was based on previous studies in which DEX protected the kidney (Si et al., 2013; Li et al., 2018; Qiu et al., 2018). In the present study, ATI was used to confirm that DEX exerts its pharmacological role through the α_2_-AR. However, we were still perplexed with regard to the potential mechanism by which DEX upregulates autophagy.

According to previous studies, mTOR is one of the most important negative regulators of autophagy. In addition, AMPK can reportedly upregulate autophagy by suppressing mTOR phosphorylation. The AMPK/mTOR signaling pathway has emerged as a crucial regulator of autophagy (Kim et al., 2011; Kim et al., 2016). AMPK activation generally plays a protective role in various renal injury models (Allouch and Munusamy, 2017; Bao et al., 2019; Liu et al., 2019b). Recently, DEX has been confirmed to exert protective effects by activating AMPK and suppressing inflammatory responses (Wang et al., 2018b). Our results indicate that DEX pretreatment can upregulate AMPK phosphorylation and suppress mTOR phosphorylation. Above all, these results indicate that DEX can enhance autophagy through the AMPK/mTOR pathway in acute kidney injury induced by LPS.

In summary, our results suggest an effective role of DEX in protecting against LPS-induced AKI *via* inhibited inflammation. This study also provides evidence that the inflammatory response induced by NLRP3 inflammasome activation can be significantly reduced by autophagy. Finally, we confirmed that DEX enhances autophagy *via* the α_2_-AR/AMPK/mTOR pathway to inhibit activation of the NLRP3 inflammasome and subsequently alleviates LPS-induced AKI.

## Data Availability Statement

The datasets generated for this study are available on request to the corresponding author.

## Ethics Statement

The animal study was reviewed and approved by Animal Experimental Committee of Northeast Agricultural University.

## Author Contributions

HF and TY contributed to the conception and design of the study. YZ, TY, and HY conducted experiments. TY organized the database. MW performed the statistical analysis. TY wrote the first draft of the manuscript. XF and HZ wrote sections of the manuscript. All authors contributed to the article and approved the submitted version.

## Funding

This work was supported by a National Natural Science Foundation of China Grant (Grant No. 31772806); National Natural Science Foundation of China Grant (Grant No. 31802251).

## Conflict of Interest

The authors declare that the research was conducted in the absence of any commercial or financial relationships that could be construed as a potential conflict of interest.
